# RNA Polymerase II-Dependent Transcription Initiated by Selectivity Factor 1: A Central Mechanism Used by MLL Fusion Proteins in Leukemic Transformation

**DOI:** 10.3389/fgene.2018.00722

**Published:** 2019-01-14

**Authors:** Akihiko Yokoyama

**Affiliations:** Tsuruoka Meatabolomics Laboratory, National Cancer Center, Yamagata, Japan

**Keywords:** RNA polymerase, SL1, transcription, leukemia, MLL, AEP, DOT1L

## Abstract

Cancer cells transcribe RNAs in a characteristic manner in order to maintain their oncogenic potentials. In eukaryotes, RNA is polymerized by three distinct RNA polymerases, RNA polymerase I, II, and III (RNAP1, RNAP2, and RNAP3, respectively). The transcriptional machinery that initiates each transcription reaction has been purified and characterized. Selectivity factor 1 (SL1) is the complex responsible for RNAP1 pre-initiation complex formation. However, whether it plays any role in RNAP2-dependent transcription remains unclear. Our group previously found that SL1 specifically associates with AF4 family proteins. AF4 family proteins form the AEP complex with ENL family proteins and the P-TEFb elongation factor. Similar complexes have been independently characterized by several different laboratories and are often referred to as super elongation complex. The involvement of AEP in RNAP2-dependent transcription indicates that SL1 must play an important role in RNAP2-dependent transcription. To date, this role of SL1 has not been appreciated. In leukemia, AF4 and ENL family genes are frequently rearranged to form chimeric fusion genes with *MLL*. The resultant *MLL* fusion genes produce chimeric MLL fusion proteins comprising MLL and AEP components. The MLL portion functions as a targeting module, which specifically binds chromatin containing di-/tri-methylated histone H3 lysine 36 and non-methylated CpGs. This type of chromatin is enriched at the promoters of transcriptionally active genes which allows MLL fusion proteins to selectively bind to transcriptionally-active/CpG-rich gene promoters. The fusion partner portion, which recruits other AEP components and SL1, is responsible for activation of RNAP2-dependent transcription. Consequently, MLL fusion proteins constitutively activate the transcription of previously-transcribed MLL target genes. Structure/function analysis has shown that the ability of MLL fusion proteins to transform hematopoietic progenitors depends on the recruitment of AEP and SL1. Thus, the AEP/SL1-mediated gene activation pathway appears to be the central mechanism of MLL fusion-mediated transcriptional activation. However, the molecular mechanism by which SL1 activates RNAP2-dependent transcription remains largely unclear. This review aims to cover recent discoveries of the mechanism of transcriptional activation by MLL fusion proteins and to introduce novel roles of SL1 in RNAP2-dependent transcription by discussing how the RNAP1 machinery may be involved in RNAP2-dependent gene regulation.

## Eukaryotes Have Three Major RNA Polymerases

**Figure 1:**
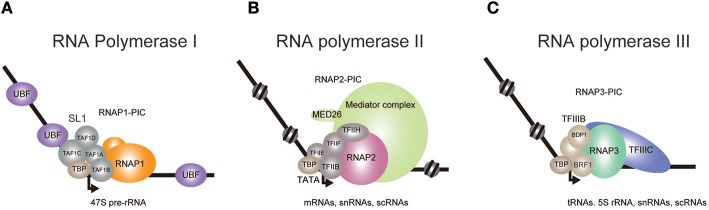
Transcriptional initiation complexes of the three eukaryotic RNA polymerases. The pre-initiation complexes (PIC) of RNA Polymerase I **(A)**, II **(B)**, and III **(C)** are depicted. **(A)** In the presence of UBF, SL1 recognizes the rDNA promoter and recruits RNAP1. TBP binding induces a bend in the DNA, which leads to the formation of PIC. **(B)** TBP binding and subsequent recruitment of RNAP2 and other auxiliary factors completes the formation of PIC. Mediator is thought to be involved in PIC stabilization and in the initiation of transcription. **(C)** At tRNA promoters, TFIIIC located in the intragenic region recruits TFIIIB, which contains BDP1 and BRF1, to further recruit RNAP3.

In prokaryotes, one RNA polymerase transcribes all genes. Eukaryotic cells contain multiple RNA polymerases, which transcribe different classes of genes (Roeder and Rutter, 1969; Thomas and Chiang, 2006; White, 2008; Vannini and Cramer, 2012; Turowski and Tollervey, 2016; Khatter et al., 2017; Zhang et al., 2017). Most genes are transcribed by three major RNA polymerases, RNA Polymerase I, II, and III (RNAP1, RNAP2, and RNAP3, respectively). RNAP1 transcribes pre-rRNA, which is later processed into three large rRNA species, 28S, 18S, and 5.8S. RNAP2 transcribes protein-coding genes to yield mRNAs. RNAP3 transcribes 5S rRNA and tRNAs. Small nuclear RNAs and small cytoplasmic RNAs are transcribed by either RNAP2 or RNAP3 (Figure 1).

## RNA Polymerase I

RNAP1 synthesizes the 47S pre-rRNA, which is processed into mature 28S, 18S, and 5.8S rRNAs (Goodfellow and Zomerdijk, 2013; Khatter et al., 2017; Zhang et al., 2017). The Human genome contains ~400 rDNA repeats, about 50% of which are transcribed, accounting for up to 60% of the entire transcriptional activity (Birch and Zomerdijk, 2008; Schlesinger et al., 2009). Pre-initiation complex (PIC) formation of RNAP1 at the rDNA promoter is triggered upon association of selectivity factor 1 (SL1) with the core promoter, in the presence of UBF (Learned et al., 1985; Eberhard et al., 1993; Comai et al., 1994; Zomerdijk et al., 1994; Heix et al., 1997; Gorski et al., 2007) (Figure 1A). SL1 comprises TBP, TAF1A (also known as TAFI48), TAF1B (also known as TAFI63), TAF1C (also known as TAFI110), and TAF1D (also known as TAFI41) and recruits the RNAP1 complex to induce PIC formation. It is thought that all three polymerases employ similar mechanisms to start transcription (Naidu et al., 2011; Vannini and Cramer, 2012; Khatter et al., 2017), in which the universal role of TBP is to bend the template DNA, while TAF1B, TFIIB, and BRF1 proteins respectively recruit corresponding polymerases to the promoter.

## RNA Polymerase II

RNAP2 has been most rigorously studied and shown to collaborate with various associated factors in a step-wise manner to transcribe mRNAs (Roeder, 1996; He et al., 2013). Initiation of *in vitro* basal transcription on a model promoter starts with loading of the TATA binding protein (TBP) to the TATA box (Basehoar et al., 2004), which is positioned approximately 25 nucleotides upstream of the transcription start site (Roeder, 1996; He et al., 2013). TBP binding induces a bend in the double helix (Kim J. L. et al., 1993; Kim Y. et al., 1993) and recruits TFIIB to stabilize the DNA/protein complex (Figure 1B). TFIIB then recruits RNAP2 and TFIIF to form a PIC (Roeder, 1996; He et al., 2013). The initiation of transcription requires the recruitment of TFIIE and TFIIH. TFIIH unwinds DNA at the initiation site and phosphorylates the Ser 5 residue of the RNAP2 C-terminal domain heptapeptide repeat to release the polymerase from the PIC. *In vivo* RNAP2-dependent transcription is much more complicated. TBP binds to various TBP-associated factors (TAFs) to form a large complex called TFIID, which facilitates promoter recognition, especially at promoters lacking an obvious TATA box (Dynlacht et al., 1991; Pugh and Tjian, 1991). Gene promoters with a TATA box tend to be bound by the SAGA complex which includes TBP, SUPT3H, and GCN5 (Basehoar et al., 2004; Rodríguez-Navarro, 2009). Therefore, it was thought that TATA-containing genes were mainly regulated by the SAGA complex, while TATA-less genes were independently regulated by TFIID (Pugh and Tjian, 1991; Basehoar et al., 2004). However, recent studies in yeast indicate that most genes utilize both TFIID and SAGA, and that the relative contribution of each complex likely depends on the individual context (Baptista et al., 2017; Warfield et al., 2017). The Mediator co-activator complex is also involved in transcription initiation for the expression of nearly all genes (Malik and Roeder, 2010; Warfield et al., 2017). Mediator disruption caused more severe defects than did the disruption of TFIID subunits, suggesting that there may be a low level of TFIID-independent transcription at many genes that is derived from PICs assembled with TBP and lacking TAFs. Nearly all RNAP2-regulated genes, with or without a TATA box in the promoter, are thought to use TBP for transcriptional activation.

## RNA Polymerase III

RNAP3 transcribes 5S rRNA, tRNAs, and various small non-coding RNAs (White, 2008; Vannini and Cramer, 2012; Turowski and Tollervey, 2016; Khatter et al., 2017). The clearest feature of RNAP3 transcripts is that they are all untranslated and less than 300 base pairs in length. tRNA gene transcription requires TFIIIB and TFIIIC (Figure 1C). TFIIIC binds to intragenic elements and positions TFIIIB onto the tRNA promoter. TFIIIB, which contains TBP and BRF1, then induces PIC formation to start RNAP3 transcription.

## RNA Polymerase I/SL1-Dependent ribosomal RNA Transcription in Cancer

In cancer cells, rRNA transcription is upregulated. This increases the cell's ability to produce proteins to meet the metabolic demands of quickly proliferating cancer cells (White, 2008). High level pre-rRNA expression is observed in cancer cells and is correlated with tumor stage (Williamson et al., 2006). MYC is highly expressed in most cancer cells and upregulates cell cycle-related genes and metabolism-related genes to promote cell division and anabolism (Dang, 2012). MYC also activates rRNA transcription directly (Arabi et al., 2005; Grandori et al., 2005; Shiue et al., 2009) and indirectly by activating UBF expression (Poortinga et al., 2004). PTEN, which is often inactivated in cancer, represses RNAP1-dependent transcription by disrupting the SL1 complex (Zhang et al., 2005). Thus, loss of PTEN facilitates cancer specific metabolism in part by enhancing SL1-mediated rRNA transcription.

## AF4 Family/ENL Family/P-TEFb Complex

**Figure 2:**
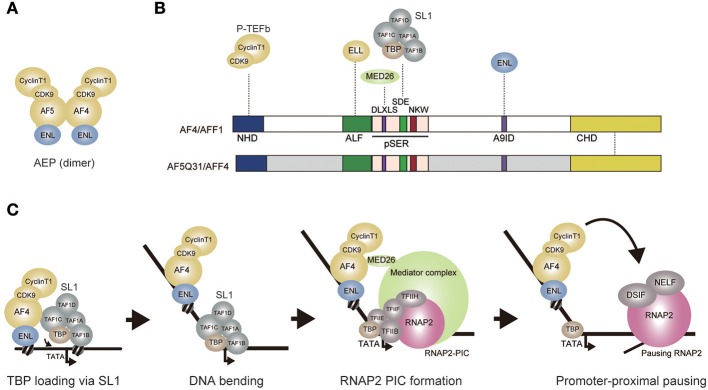
AEP-dependent transcriptional activation. **(A)** Composition of the AEP complex (AF4 family/ENL family/P-TEFb complex). AF4 and AF5q31 can form a tero dimer and associate with ENL, CyclinT1, and CDK9. **(B)** Schematic representation of the AF4 and AF5Q31 protein structures. Protein-protein interaction is shown by a dotted line. NHD, N-terminal homology domain; ALF, AF4/LAF4/FMR2 homology domain; pSER, poly-serine. A9ID, AF9 interaction domain; CHD, C-terminal homology domain; **(C)** A hypothetical model of AEP-dependent transcriptional activation. First, AEP recruits SL1 and loads TBP onto the promoter through the SDE and NKW motifs. TBP loading induces a bend. SL1 is then dismantled while TFIIB replaces TAF1B. AEP recruits Mediator through MED26 to facilitate transcription initiation. P-TEFb activates transcription elongation of the RNAP2 complex paused by negative elongation factors such as DSIF and NELF. DSIF: DRB sensitivity inducing factor. NELF: Negative elongation factor.

Unexpectedly, our group identified SL1 as a specific interactor of AF4 (also known as AFF1), which is involved in RNAP2-dependent transcriptional activation (Okuda et al., 2015). This result indicated that SL1 is involved in both RNAP1- and RNAP2-dependent transcription. AF4 is a member of the AF4 protein family that is composed of AF4, AF5Q31 (also known as AFF4), LAF4 (also known as AFF3), and FMR2 (also known as AFF2). Previously, we purified a protein complex nucleated by AF4 and identified AF5Q31, ENL (also known as MLLT1), CDK9, and CyclinT1 (also known as CCNT1) as its components (Yokoyama et al., 2010) (Figure 2A). CDK9 and CyclinT1 form a complex called P-TEFb, which activates transcription elongation by phosphorylation of the RNAP2 complex paused by negative elongation factors such as DSIF an NELF (Wada et al., 1998a,b; Yamaguchi et al., 1999; Peterlin and Price, 2006). We named this complex the AF4 family/ENL family/P-TEFb complex (AEP) (Yokoyama et al., 2010). ELL, which retains transcription elongation activity (Shilatifard et al., 1996), was also shown to associate with the AF4 family protein and other AEP components, and this complex is often referred to as the super elongation complex (Lin et al., 2010). Similar complexes were independently identified and characterized in several laboratories and have been shown to activate the RNAP2-dependent transcription elongation step for a subset of genes including heat shock protein genes and the HIV viral genome (He et al., 2010; Lin et al., 2010; Sobhian et al., 2010). Therefore, it is thought that AEP activates transcription by activating transcription elongation (Luo et al., 2012).

## AF4 Family Proteins Activate Transcription Via SL1

AF4 family proteins have been shown to activate RNAP2-dependent transcription (Prasad et al., 1995; Ma and Staudt, 1996; Morrissey et al., 1997; Hillman and Gecz, 2001). GAL4-dependent transactivation assays use reporter gene expression to indicate activation of transcription. Reporter gene transcription, such as that of the firefly luciferase gene, is driven by a minimum promoter tethered to multiple GAL4 responsive elements and is measured in the presence of the GAL4 DNA binding domain fused with the domain being tested. Using this approach, the serine-rich pSER domain of AF4 protein family (Nilson et al., 1997) was shown to activate transcription (Okuda et al., 2015) (Figure 2B). In contrast, modules presumed to activate transcription elongation by recruiting P-TEFb or ELL, did not exhibit transactivation activity. These results indicate that, in addition to recruiting elongation factors, AF4 family proteins possess transcriptional activation functions. Assuming that the pSER domain associates with transcriptional coactivators, our group purified proteins associated with the GAL4-pSER fusion protein and identified SL1 as a specific pSER domain binding factor. Chromatin immunoprecipitation coupled with deep sequencing (ChIP-seq) analysis in HEK293T cells showed that TAF1C of SL1 co-localizes with AEP components in the vicinity of transcription start sites of AEP target genes that are transcribed by RNAP2 (Okuda et al., 2017). Knockdown of TAF1C resulted in decreased expression of AEP target genes (defined as the genes whose expression is reduced by ENL knockdown) (Okuda et al., 2015). Deletion of the TATA box in the luciferase reporter cassette resulted in loss of pSER domain-mediated transactivation. Taken together, these results suggest that the pSER domain first recruits SL1 and then loads TBP to the TATA box to activate RNAP2-dependent transcription initiation.

## MED26 Potentiates AF4-Dependent Transcription Initiation by SL1

RNAP2 PIC formation is facilitated by Mediator, a large protein complex composed of ~30 subunits (Malik and Roeder, 2010). Mediator exists in a variety of subunit compositions, most of which are conserved from yeast to metazoans. MED26, one of a few metazoan-specific Mediator subunits, was shown to associate with AEP in addition to associating with other Mediator complex components (Takahashi et al., 2011). The pSER domain can be divided into three subdomains, each of which contains one evolutionarily conserved motif, DLXLS, SDE, and NKW (Okuda et al., 2016) (Figure 2B). The DLXLS motif is a binding platform for MED26 while the SDE motif is responsible for binding to SL1. Although the function of the NKW motif remains unclear, it is required for transactivation and is therefore postulated to play a role in RNAP2-dependent transcription processes (Okuda et al., 2015). The SDE and NKW motifs are necessary and sufficient to activate transcription in GAL4-dependent transactivation assay. The DLXLS motif is dispensable but can enhance SL1-mediated transcription, presumably by recruiting Mediator (Okuda et al., 2016). These results indicate that AEP activates transcription initiation primarily via SL1, which can be further potentiated by Mediator. This is contradictory to the current view that AEP is specialized to transcription elongation. Hence, I propose that AEP is a multi-functional transcriptional machinery that can activate both initiation and elongation of transcription (Figure 2C).

## AEP-dependent Transactivation is the Central Mechanism Used by MLL Fusion Proteins in Leukemogenesis

**Figure 3:**
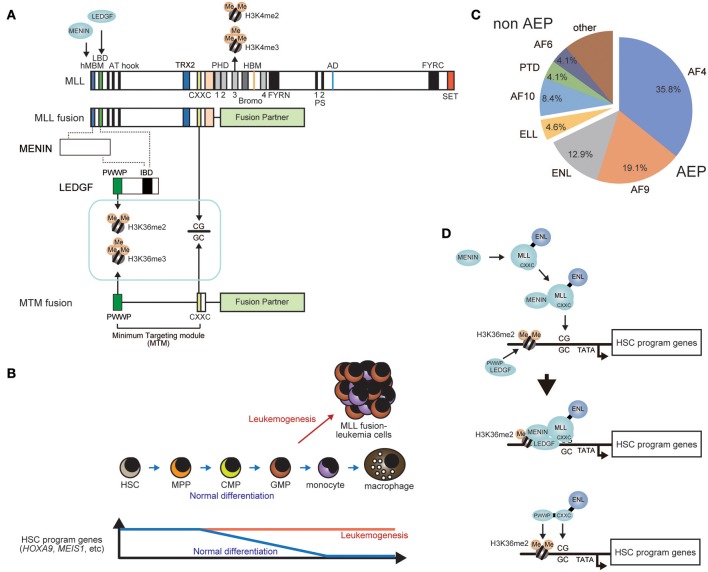
General mechanism of leukemogenesis by MLL fusion proteins. **(A)** Schematic representation of the structures of wild type MLL and the MLL fusion protein. The PWWP domain of LEDGF recognizes di-/tri-methylated histone H3 lysine 36. The CXXC domain of MLL binds to unmethylated CpGs. The PHD finger 3 binds to di-/tri-methylated Histone H3 lysine 4 (H3K4me2/3). The minimum targeting module necessary for target recognition (MTM) comprises the PWWP and CXXC domains. HBM, HCF binding motif; PS, processing site; AD, activation domain; FYRN, FY-rich domain N-terminal; FYRC, FY-rich domain C-terminal. **(B)** Constitutive activation of HSC program genes in leukemogenesis. Expression of HSC program genes progressively decreases during normal differentiation. However, MLL fusion proteins constitutively activate HSC program genes to cause leukemia. **(C)** Ratio of fusion partners in MLL-r leukemia cases. Relative frequency of each fusion partner is shown in a pie chart (adopted from the report from Meyer et al., 2018). AEP components such as AF4, AF9, and ENL account for two-thirds of MLL-r leukemia. **(D)** A model of target recognition by MLL fusions proteins. First, the MLL fusion protein associates with MENIN, while LEDGF binds to nucleosomes containing H3K36me2/3 marks. Next, the MLL fusion/ MENIN complex forms a stable complex with LEDGF on the promoter of HSC program genes. The MTM-ENL fusion protein binds the same promoters as MLL fusion proteins.

AEP components are frequent targets for chromosomal translocation with the *MLL* gene (also known as *KMT2A, HRX, MLL1, HTRX*, and *ALL1*) (Ziemin-van der Poel et al., 1991; Djabali et al., 1992; Tkachuk et al., 1992; Nakamura et al., 2002; Li and Ernst, 2014; Winters and Bernt, 2017; Yokoyama, 2017). MLL is a transcriptional regulator that retains transcriptional activation activity and histone methyltransferase (HMT) activity and involved in transcriptional maintenance of Homeobox (HOX) genes (Zeleznik-Le et al., 1994; Yu et al., 1995, 1998; Ernst et al., 2001; Milne et al., 2002; Nakamura et al., 2002). The resultant MLL-AEP fusion proteins cause aggressive acute leukemia (Figure 3A) (Krivtsov and Armstrong, 2007; Li and Ernst, 2014; Winters and Bernt, 2017). Leukemia involving MLL gene rearrangements (MLL-r leukemia) is the cause of 5–10% of all acute leukemia cases (Meyer et al., 2018) and is generally associated with poor prognosis (Rowley, 2008). MLL-r leukemia cells express a subset of genes including *HOXA9* and *MEIS1* whose expression is normally confined to immature hematopoietic cells such as hematopoietic stem cells (HSCs) (hereafter we refer to as HSC program genes) (Armstrong et al., 2002; Yeoh et al., 2002; Krivtsov et al., 2006; Somervaille and Cleary, 2006) (Figure 3B). Sustained expression of *HOXA9* and *MEIS1* in hematopoietic progenitors causes leukemia in mouse models (Kroon et al., 1998), suggesting that these two genes are strong drivers of leukemogenesis. MLL fusion proteins directly bind the promoters of these HSC program genes and constitutively activate their transcription (Ayton and Cleary, 2003; Somervaille and Cleary, 2006; Garcia-Cuellar et al., 2016; Kerry et al., 2017; Okuda et al., 2017). Thus, the MLL fusion protein is a constitutively-active transcriptional machinery that transforms hematopoietic progenitors by aberrantly activating HSC program genes (Krivtsov et al., 2006; Yokoyama, 2017). Although *MLL* fuses with more than 100 different fusion partners (Meyer et al., 2018), AEP components constitute two-thirds of MLL-r leukemia cases (Figure 3C), indicating that merging the functions of MLL and AEP is the most efficient way to generate powerful leukemic oncogenes. Among the AEP components, AF4 is the most frequent fusion partner for MLL, while AF5Q31 and LAF4 also fuse with MLL in rare cases of leukemia (Ma and Staudt, 1996; Taki et al., 1999; Meyer et al., 2018). The second most frequent fusion partner is AF9 (also known as MLLT3) (Meyer et al., 2018), which is a homolog of ENL. ENL and AF9 constitute the ENL protein family and form a fusion with MLL in one-third of MLL-r leukemia cases. ELL, which also binds to the AF4 family protein, fuses with MLL in leukemia (DiMartino et al., 2000; Luo et al., 2001; Lin et al., 2010). These results strongly indicate that the transcriptional activation function of AEP is the central mechanism utilized by MLL fusion proteins in leukemogenesis.

## Target Chromatin of MLL and MLL Fusion Proteins

The role of the MLL portion of MLL fusion proteins is mainly target recognition. Genome-wide ChIP-seq analysis showed that MLL fusion proteins bound to the target chromatin of wild-type MLL near transcription start sites (Guenther et al., 2005, 2008; Wang et al., 2011; Okuda et al., 2017). Most of the target genes bound by MLL fusion proteins are included within the list of MLL target genes (Milne et al., 2005; Wang et al., 2011), and some genes are exclusively regulated by wild-type MLL (Artinger and Ernst, 2013; Li et al., 2013). Wild type MLL retains various chromatin reader modules including plant homeodomain (PHD) fingers and a Bromodomain, which are lost in MLL fusion proteins (Figure 3A). Thus, the mode of target recognition is expected to be somewhat different between wild type MLL and MLL fusion proteins. For example, PHD finger 3 has been shown to bind to di-/tri-methylated histone H3 lysine 4 (H3K4me2/3) (Wang et al., 2010), which plays a significant role in target recognition by wild type MLL (Milne et al., 2010). These observations indicate that the MLL portion retained by MLL fusion proteins confers the ability to recognize a subset of, but not all of, wild-type MLL target genes. Whether the presence of wild type MLL is required for MLL fusion-dependent leukemic transformation has been a topic of discussion. One study showed that the remaining wild type allele is required for leukemogenesis (Thiel et al., 2010). But recently, another study showed that MLL is dispensable, while MLL2 (also known as KMT2B), the closest homolog of MLL, plays a major role in sustaining leukemogenesis (Chen et al., 2017), indicating complex redundancy and independency within the target genes of MLL fusion proteins, MLL and MLL2.

## Mechanism of Target Recognition by MLL Fusion Proteins

The structural requirements of MLL fusion-dependent leukemic transformation can be evaluated using the *ex vivo* myeloid progenitor transformation assay (Lavau et al., 1997). In this assay, a retrovirus carrying an MLL fusion gene is transduced into murine bone marrow-derived immature hematopoietic progenitors and the cells are cultured *ex vivo* in semi-solid media containing the required cytokines. Transduction of a functional MLL fusion gene results in the cells expressing high *Hoxa9* levels and continuing to proliferate after rounds of replating, while non-transduced cells stop proliferating during early passages (Ayton and Cleary, 2003). Transformed cells can be cultured for more than 5 months and are considered “immortalized.” Immortalization is an important feature of leukemic transformation and reflects the aberrant self-renewal of cancer cells. Using this assay, domains within the MLL-ENL fusion that are required for transformation were identified (Lavau et al., 1997; Slany et al., 1998; Ayton et al., 2004). The N-terminal region upstream of the AT hooks was shown to be required for transformation (Figure 3A). This region contains a motif responsible for the strong association with MENIN (hMBM: the high affinity MENIN binding motif) (Yokoyama et al., 2004, 2005). MLL-MENIN association triggers further association with LEDGF through the LEGDF binding domain (LBD) (Yokoyama and Cleary, 2008; Huang et al., 2012). LEDGF contains the PWWP domain, which specifically binds the nucleosomes with di-/tri-methylated histone H3 lysine 36 (H3K36me2/3) (Eidahl et al., 2013; Okuda et al., 2014; Zhu et al., 2016) (Figure 3D). Mutant constructs of MLL-ENL lacking the hMBM or the LBD failed to transform myeloid progenitors because sequential association of MENIN and LEDGF is critical for leukemic transformation. However, an artificial construct tethering the PWWP domain to the MLL-ENL mutant lacking the hMBM transformed myeloid progenitors, indicated that MENIN's primary role is to incorporate the PWWP domain into the MLL-ENL complex and that other structures of MENIN and LEDGF are dispensable (Yokoyama and Cleary, 2008). Further structure/function analysis demonstrated that only three domains of the MLL-ENL complex are required for leukemic transformation: the PWWP domain of LEDGF, the CXXC domain of MLL, and the ENL portion (Okuda et al., 2014) (Figures 3A,D). The CXXC domain specifically binds to unmethylated CpGs (Birke et al., 2002; Allen et al., 2006; Cierpicki et al., 2010). Because an artificial construct composed of the PWWP and CXXC can target the promoters of HSC program genes, combination of these two domains is defined as the minimum targeting module (MTM) (Figure 3A).

## Di-/Tri-methylated Histone H3 Lysine 36

Many epigenetic modifiers possess PWWP domains. For example, BRPF1 has a PWWP domain, which also binds to H3K36me2 and H3K36me3 and can functionally substitute for that of LEDGF, indicating that the PWWP domain is a chromatin reader module for H3K36me2/3 in general (Vezzoli et al., 2010; Okuda et al., 2014). H3K36me2 marks are found in active gene promoters and are introduced by histone methyl transferases (HMTs) like ASH1L and NSD2 (Kuo et al., 2011; Zhu et al., 2016). When transcription is ongoing, further methylation on an H3K36me2 mark in the gene body region occurs to produce H3K36me3 by another HMT termed SETD2 complexed with elongating RNAP2 (Wagner and Carpenter, 2012). This H3K36me3 modification highlights transcribed regions and is required for efficient DNA damage response (Mar et al., 2017). Therefore, heterozygous loss of SETD2, which leads to blunt DNA damage response against chemotherapy, is often found in relapsed leukemia (Mullighan et al., 2011; Mar et al., 2014; Xiao et al., 2016). SETD2 was recently reported to physically interact with MLL fusion proteins and may also be implicated in the efficient targeting of MLL fusion proteins to the target promoters (Skucha et al., 2018).

## Unmethylated CG DNA Sequence

Unmethylated CG DNA sequence, which are specifically recognized by the CXXC domain, are enriched in gene promoters, and are linked to transcription initiation (Cedar and Bergman, 2009; Bird, 2011). If the cytosine of CpGs is methylated, the CXXC domain no longer binds to the CG sequence (Allen et al., 2006). Unmethylated CpGs are an epigenetic mark of non-silenced promoters because methylation of CpGs in the promoter are associated with transcriptional silencing. Through the PWWP and CXXC domains, the MLL fusion complex targets transcriptionally-active CpG-rich promoters (Figure 3D). During embryogenesis, MLL maintains segment-specific expression of *HOX* genes (Yu et al., 1995). *HOX* genes are called “cellular memory” genes as their position-specific expression patterns are maintained during development (Deschamps and van Nes, 2005; Wang et al., 2009). MLL is not required for initial activation of *HOX* gene expression, but is required for the maintenance of *HOX* gene expression (Yu et al., 1998), indicating that MLL is involved in maintaining an established expression pattern, rather than in determining the expression pattern itself. Therefore, it is likely that MLL targets CpG-rich promoters which were previously transcriptionally-active in the maternal cell and re-activates transcription in daughter cells to maintain the *HOX* gene expression patterns.

## Additional Mechanisms of Target Recognition by MLL Fusion Proteins

Resent research showed that the target chromatin of MLL fusion proteins is not confined to the promoter region. Localization of MLL fusion proteins spreads into the gene body at some MLL target genes, which are often hypo-methylated and highly transcribed (Kerry et al., 2017). It has been suggested that this aberrant localization is implicated in disease progression. Moreover, in some genes MLL-ENL localizes near the transcription end site and activates gene expression predominantly at transcription elongation levels (Garcia-Cuellar et al., 2016). These results suggest that MLL fusion proteins can be involved in multiple facets of gene activation through binding outside of the promoter region even though their primary targets are CpG-rich promoter regions. Moreover, the structure juxtaposed to the CXXC domain of MLL has been shown to associate with the PAF1 complex, which is thought to bind elongating RNAP2 (Milne et al., 2010; Muntean et al., 2010). PAF1 association may be involved in the fine-tuning of target recognition and/or noncanonical targeting mechanisms mentioned above.

## SL1-Mediated Transcriptional Activation by MLL Fusion Proteins

**Figure 4:**
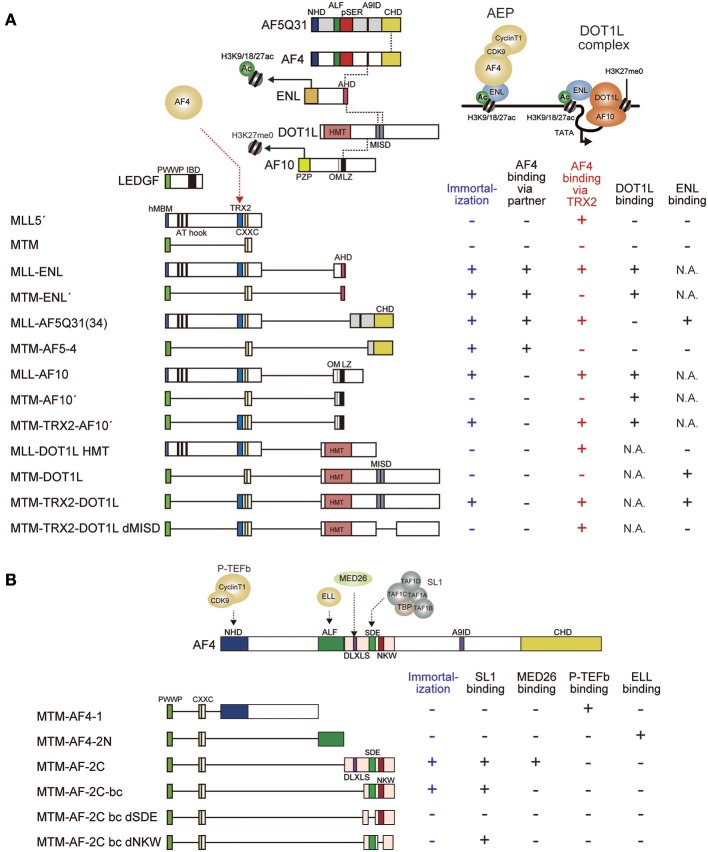
Structural requirements of MLL fusion proteins for leukemic transformation. **(A)** Schematic representation of the structures of various MLL fusion constructs. MLL fusion proteins recruit AF4 and ENL through different mechanisms to immortalize hematopoietic progenitors. The minimal construct of MLL-ENL which immortalizes hematopoietic progenitors (MTM-ENL') is composed of MTM and the AHD of ENL, while that of MLL-AF5Q31(MTM-AF5-4) is composed of MTM and the CHD of AF5Q31, indicating that recruitment of AF4 to the MLL target chromatin confers transforming activity. By contrast, the minimal transforming construct of MLL-AF10 (MTM-TRX2-AF10') is composed of MTM, the TRX2 domain, and the OMLZ domain of AF10, indicating that MLL-AF10 requires the TRX2 domain for AF4 recruitment and the OMLZ domain for ENL recruitment. In concordance with this notion, the MTM-TRX2-DOT1L construct immortalizes hematopoietic progenitors whereas deletion of the ENL binding domain (MISD) results in loss of transformation. Dotted lines indicate protein-protein interaction. Associated properties of each construct, such as the ability to immortalize myeloid progenitors, and the binding abilities to AF4, DOT1L, and ENL are shown on the right. Immortalizing ability and AF4 binding ability through the TRX2 domain are highlighted in blue and red, respectively. MISD: minimum interaction site for DOT1L. One MISD, located at the residues 628–653, was omitted because of its very weak affinity (Kuntimaddi et al., 2015). N.A., not applicable. **(B)** Schematic representation of the structures of various MTM-AF4 fusion constructs. MTM-AF4 fusion proteins recruit SL1 through the SDE motif and activate transcription through the NKW motif to immortalize hematopoietic progenitors. Binding modules for P-TEFb or ELL did not confer transforming ability. Associated properties of each construct, including the ability to immortalize myeloid progenitors, binding abilities to SL1, MED26, P-TEFb, and ELL are shown on the right.

**Figure 5:**
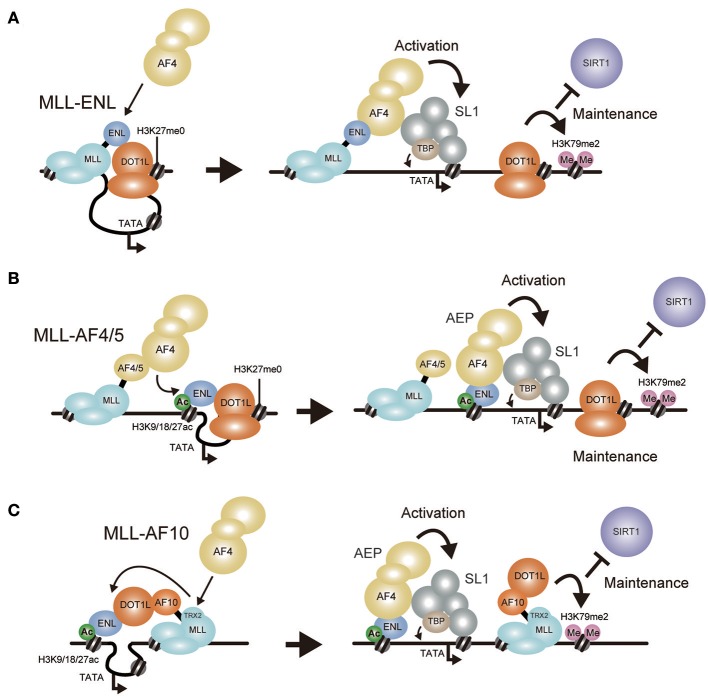
Models of MLL fusion-dependent gene activation. Working models of gene activation by MLL-ENL **(A)**, MLL-AF4/AF5Q31 **(B)**, and MLL-AF10 **(C)** are shown. **(A)** MLL-ENL associates with the DOT1L complex on the target chromatin. Because AF4 binds ENL with higher affinity than DOT1L, MLL-ENL preferentially forms a complex with AEP. AEP components recruit SL1 to activate transcription while the DOT1L complex maintains transcriptionally-active chromatin by methylating histone H3 lysine 79 to repel SIRT1 transcriptional repressor. **(B)** MLL-AF4/AF5q31 recruits AF4 proteins, which subsequently bind to ENL tethered to chromatin containing histone H3 K9/18/27ac marks. AEP activates transcription via SL1. **(C)** MLL-AF10 forms a complex with DOT1L and ENL on the target chromatin. AF4 proteins are recruited via the TRX2 domain, which then bind to ENL tethered to chromatin containing histone H3 K9/18/27ac marks to form AEP.

While MLL fusion proteins target previously-active CpG-rich promoters through their MLL portions, the fusion partner portion confers the ability to activate transcription. The minimum domains within the fusion partner portion required for transformation have been identified for MLL-ENL and MLL-AF5Q31 (Slany et al., 1998; Yokoyama et al., 2010; Okuda et al., 2015). The ANC1 homology domain (AHD) of ENL and the C-terminal homology domain (CHD) of AF5Q31 are responsible for *Hoxa9* transcriptional activation and for the transformation of myeloid progenitors (Figure 4A). These domains are the binding platforms for AF4, suggesting that aberrant recruitment of AF4 to MLL target promoters is essential for MLL fusion-dependent transformation. Therefore, functional modules retained within the AF4 portion were thought to be responsible for transformation. An artificial construct in which the pSER domain of AF4 is tethered to the MTM activated *Hoxa9* expression and immortalized myeloid progenitors (Figure 4B), indicated that recruitment of the SL1 complex to MLL target promoters is necessary and sufficient for transformation. The minimum structure required for transformation was the region encompassing the SDE motif and the NKW motif, which recruit SL1 and activate transcription (Okuda et al., 2015, 2016). These results suggest that the MLL fusion proteins transform myeloid progenitors via SL1-mediated transcriptional activation through AF4. Surprisingly, other functional modules such as the N-terminal homology domain (NHD), which recruits P-TEFb, and the AF4/LAF4/FMR2 homology (ALF) domain (Nilson et al., 1997), which recruits ELL, were dispensable despite the much anticipated significance of these elongation factors. Taken together, these observations suggest that the major role of MLL fusion proteins in leukemic transformation is not to activate transcription elongation but to activate transcription initiation via SL1. Similarly, the DLXLS motif, which recruits Mediator, was also dispensable for transformation (Okuda et al., 2016), suggesting that direct recruitment of Mediator by the MLL fusion complex appears not critical either. Hence, SL1-mediated transcriptional activation by RNAP2 is the rate-limiting step for MLL fusion-dependent gene activation (Figures 5A,B).

## Maintenance of MLL Fusion-Dependent Transcription by DOT1L

Transcriptional maintenance is also required for MLL fusion-mediated leukemogenesis. MLL target genes are prone to gene silencing by transcriptional repressors such as SIRT1 histone deacetylase (Chen C. W. et al., 2015). To maintain gene expression, MLL fusion proteins utilize the DOT1L HMT. DOT1L is an epigenetic modifier that produces mono-, di-, and tri-methylated histone H3 lysine 79 marks (H3K79me1/2/3) (Feng et al., 2002; Jones et al., 2008). DOT1L forms a complex with AF10 family proteins (AF10/AF17) and ENL family proteins (ENL/AF9) (Okada et al., 2005; Mueller et al., 2007; Mohan et al., 2010) (Figure 4A). The association of DOT1L with AF10 increases DOT1L HMT activity (Deshpande et al., 2014). The AF10 family genes also form a MLL fusion gene to cause leukemia (DiMartino et al., 2002). MLL-ENL and MLL-AF10 directly recruit DOT1L to the target promoters, suggesting that aberrant DOT1L recruitment contributes to leukemogenesis (Okuda et al., 2017). Moreover, MLL fusion-transformed myeloid progenitors lose their clonogenicity upon acute loss of the *Dot1l* gene (Chang et al., 2010; Bernt et al., 2011; Jo et al., 2011; Nguyen et al., 2011; Chen et al., 2013). These observations indicated that the continuous presence of DOT1L is required for leukemic transformation and led to the development of a DOT1L HMT inhibitor for the treatment of MLL-r leukemia (Daigle et al., 2011). EPZ-5676 (Pinometostat), a potent DOT1L inhibitor, showed remarkable efficacy in rodent xenograft models (Daigle et al., 2013), confirming that DOT1L-dependent transcriptional maintenance is required for MLL fusion proteins. However, the mode of DOT1L recruitment is somewhat controversial. Some studies suggest that AF4 proteins form a stable complex with DOT1L (Bitoun et al., 2007; Lin et al., 2016). Our biochemical data suggest that AF4 family proteins do not directly associate with DOT1L (Yokoyama et al., 2010). AF4 family proteins and DOT1L bind to the AHD of ENL family proteins in a mutually exclusive manner (Mueller et al., 2007; Yokoyama et al., 2010; Okuda et al., 2017). Structural studies showed that similar hydrophobic motifs in DOT1L and AF4 bind to the same groove-like structure in AHD (Leach et al., 2013; Shen et al., 2013; Kuntimaddi et al., 2015). Therefore, AF4-ENL association and DOT1L-ENL association via AHD should be mutually exclusive. I postulate that AF4 proteins cannot form a complex with DOT1L because of this structural restraint, but do not exclude the possibility that AF4 and DOT1L can be tethered in alternative indirect manners.

## Mechanisms of Gene Activation by MLL-ENL and MLL-AF10

The minimum domain structure of the MLL portion required for transformation by MLL-AF10 differs from that required by MLL-ENL (Figure 4A). As for MLL-ENL, the MTM and the AHD, which are sufficient to recruit both AF4 and DOT1L to the MLL target genes, confer transforming ability (Okuda et al., 2014, 2015). In contrast, the MTM fused with the OMLZ domain of AF10 (MTM-AF10′), which recruits DOT1L but not AF4, exhibited relatively high *Hoxa9* expression in first round colonies but could not maintain its expression for a longer period and was unable to immortalize myeloid progenitors (Okuda et al., 2017). This indicated that DOT1L recruitment to the MLL-target promoter upregulates target gene expression insufficiently for immortalization. However, incorporation of the TRX2 domain of MLL into this MTM-AF10′ fusion construct (MTM-TRX2-AF10′) resulted in full transformation, indicating that the TRX2 domain confers additional functions to achieve full leukemic transformation. Given that one function retained by the AHD, but missing from the OMLZ domain, is the ability to recruit AF4 family proteins, we examined whether the TRX2 domain associates with AF4 family proteins on chromatin. To this end, we used the fractionation-assisted chromatin immunoprecipitation (fanChIP) method (Okuda et al., 2014), which enables us to capture protein complexes bound to chromatin. Indeed, the TRX2 domain associated with AF4 proteins (Okuda et al., 2017). Thus, MLL-AF10 recruits AF4 and DOT1L through the TRX2 domain and the OMLZ domain, respectively, to immortalize hematopoietic progenitors (Figure 5C).

Moreover, artificial MLL-DOT1L constructs exhibited similar structural requirement (Figure 4A). An artificial construct in which the MTM is fused to the entire *DOT1L* coding sequence (MTM-DOT1L), exhibited the same compromised transforming property as the MTM-AF10′ construct, while incorporation of the TRX2 domain (MTM-TRX2-DOT1L) conferred full transforming ability. These results confirmed that recruitment of both AF4 and DOT1L is required for MLL-AF10-dependent transformation. Deletion of the ENL binding domains (MISD: the minimum interaction site of DOT1L) from the MTM-TRX2-DOT1L construct (MTM-TRX2-DOT1L dMISD) resulted in loss of transformation, indicating that the ENL-DOT1L association is required for MLL-AF10-dependent transformation. This result contradicts a previous report which showed that an artificial fusion of MLL and the HMT domain of DOT1L (MLL-DOT1L HMT), lacking the ENL binding domains, transformed myeloid progenitors in a similar setting (Okada et al., 2005). However, we consistently obtain no transformation readout using this MLL-DOT1L HMT construct (Yokoyama et al., 2010), supporting our conclusion that simply recruiting DOT1L HMT activity to the MLL target chromatin is insufficient for leukemic transformation. Based on these results, I propose a model in which MLL-AF10 promotes AEP formation on nearby chromatin through AF4 recruitment by the TRX2 domain and ENL recruitment by DOT1L (Figure 5C). Thus, both MLL-ENL and MLL-AF10 appear to activate transcription in an AEP/SL1-dependent manner.

## The Role of TRX2 Domain of MLL

It is unclear how AF4 proteins associate with the TRX2 domain of MLL. Interaction between MLL and AF4 was not detected in conventional IP analysis (Yokoyama et al., 2010). Thus far, this association has only been detected in the chromatin context (Okuda et al., 2017). It is possible that some other chromatin-bound factors mediate the interaction between MLL and AF4 through the TRX2 domain. It has been reported that the region containing the TRX2 domain also associates with SHARP1, which may be involved in interaction between MLL and AF4 (Numata et al., 2018). Although the MLL 5′ portion retains the TRX2 domain, it cannot activate transcription of *Hoxa9* and transform myeloid progenitors without its fusion partner portion (Lavau et al., 1997; Slany et al., 1998; Okuda et al., 2017), indicating that AF4 bound with the TRX2 domain is transcriptionally inactive and needs to form an AEP complex with ENL on nearby chromatin to become functional (Figure 5C). Supporting this hypothesis, ENL knockdown in MLL-AF10-transformed cells resulted in loss of colony forming ability (Okuda et al., 2017).

## Mechanism of Target Recognition by the DOT1L Complex

Whether DOT1L must be directly recruited by MLL fusion proteins also remains unclear. MLL-ENL directly associates with DOT1L and AF4 through the AHD (Mueller et al., 2007; Yokoyama et al., 2010; Leach et al., 2013; Kuntimaddi et al., 2015). ChIP-seq analysis of HB1119 cells (which express MLL-ENL) showed remarkable overlap of the ChIP-signals of MLL-ENL, AF4, and DOT1L (Okuda et al., 2017). However, it is unclear whether MLL-AF4 directly recruits DOT1L to the target chromatin. Several reports have demonstrated interaction between DOT1L and MLL-AF4 or AF4 by immunoprecipitation (Bitoun et al., 2007; Lin et al., 2016). Moreover, H3K79me2 marks produced by DOT1L are associated with MLL-AF4 target genes (Krivtsov et al., 2008; Kerry et al., 2017), suggesting a mechanism that MLL-AF4 might directly recruit DOT1L to its target chromatin. However, our biochemical data demonstrated that AF4 proteins do not form a stable complex with DOT1L (Yokoyama et al., 2010; Okuda et al., 2017). This indicates that DOT1L may autonomously target similar chromatin to that targeted by MLL and AEP without the help of MLL fusion proteins. The DOT1L complex retains its own chromatin binding modules. AF10 family proteins specifically bind to unmodified histone H3 lysine 27 (H3K27 me0) through their PHD finger-Zn knuckle-PHD finger (PZP) domain (Chen S. et al., 2015) (Figure 4A). The YEATS domain of ENL binds to acetylated histone H3 lysine 9/18/27 (Li et al., 2014; Erb et al., 2017; Wan et al., 2017). With those chromatin reader modules, the DOT1L complex possibly binds to its target chromatin by itself. Because AEP and the DOT1L complex both share the ENL family proteins as a component, they should target the same chromatin containing acetylated histone H3 K9/18/27. Consistent with this hypothesis, AEP and the DOT1L complex colocalized at the promoter-proximal regions of ENL target genes in HEK293T cells (Okuda et al., 2017). Binding affinity of ENL family proteins to AF4 family proteins is stronger than that to DOT1L *in vitro* (Leach et al., 2013). Therefore, the DOT1L complex likely provides ENL family proteins to AF4 family proteins nearby. As such, the DOT1L complex promotes the chromatin association of AEP. AEP/SL1-mediated transcription may in turn stimulate recruitment of the DOT1L complex in a feedback loop mechanism as mono-ubiquitination of histone H2B, which is coupled with transcription, stimulates DOT1L-dependent methylation activity of Histone H3 (McGinty et al., 2008). Such interplay between AEP and the DOT1L complex appears to be present and needs to be investigated in more detail in the future.

## Direct Recruitment of AEP not DOT1L is Required for Leukemic Transformation by MLL Fusion Proteins

Structure/function analysis data using the myeloid progenitor transformation assay indicate that modules that recruit AEP, but not DOT1L, are necessary and sufficient for transformation by MLL fusion proteins (Figure 4A). For instance, MLL fused with the CHD of AF5Q31, which binds to AF4 but not DOT1L, transformed myeloid progenitors (Okuda et al., 2015, 2017). The *MLL-Af4* fusion gene, in which the human *MLL* gene is fused to murine *Af4* gene, was shown to transform hematopoietic progenitors to develop leukemia *in vivo* (Lin et al., 2016). An artificial construct in which MLL is fused to the CHD portion of murine AF4 successfully induced leukemia *in vivo* (Lin et al., 2017). These observations favor the model that direct recruitment of AF4 family proteins but not DOT1L is the critical step for MLL fusion-dependent gene activation, and that the DOT1L complex targets to similar chromatin autonomously to maintain transcription (Figure 5B). Formation of the MLL fusion/MENIN complex can be inhibited by a small compound (MI-2, MI-2-2, MI-463, MI-503) (Grembecka et al., 2012; Shi et al., 2012). Simultaneous inhibition of MENIN-MLL interaction and DOT1L HMT activity synergistically induces differentiation of MLL fusion-associated leukemia cells (Dafflon et al., 2017; Okuda et al., 2017), supporting the notion that the MLL fusion complex and the DOT1L complex collaborate to induce leukemia.

## How Does SL1 Activate RNAP2-Dependent Transcription?

Multiple lines of evidence support the notion that SL1 is involved in AEP-dependent gene activation. For example, genome-wide ChIP-seq analysis demonstrated that TAF1C co-localizes with AF4 and RNAP2 at transcription start sites (Okuda et al., 2015, 2017). *Taf1c* knockdown causes down-regulation of AEP-target genes in mouse embryonic fibroblasts (Okuda et al., 2015). Moreover, the pSER domain, which is the binding platform for SL1, can be functionally replaced by a transcriptional activation domain for RNAP2. However, the precise mechanism by which SL1 activates RNAP2-dependent transcription is still unknown. A GAL4-pSER fusion protein activates RNAP2-dependent transcription on an artificial GAL4-responsive promoter containing a TATA box. This activation can be abolished if the TATA box is removed, indicating that the pSER domain promotes loading of TBP onto the TATA box in the form of SL1. After loading of TBP, SL1 must be dismantled and then TAF1B needs to be replaced by TFIIB, which share structural and functional similarities (Naidu et al., 2011). This leads RNAP2 to form an RNAP2-PIC (Figure 2C). The NKW motif of the pSER domain is required for transactivation and transformation and therefore is expected to play an important role. I speculate that the NKW motif binds to SL1 to induce conformational changes that facilitate either TBP loading and/or TFIIB exchange.

## Why does MLL Fusion Prefer AEP Components as its Fusion Partner?

It is odd that MLL predominantly prefers AEP components as its fusion partner. AEP/SL1-dependent gene activation seems much less potent compared to that of other activation domains that recruit TFIID (Okuda et al., 2015). Yet, AEP components are preferentially chosen by MLL. This suggests that AEP/SL1-dependent transcriptional activation has some advantage over TFIID-dependent transcriptional activation. Transcription oscillates during the cell cycle (Gottesfeld and Forbes, 1997; Liu et al., 2017). The metaphase Cyclin/CDK complex phosphorylates SL1, which hinders the interaction between SL1 and UBF, to shut off rRNA transcription during metaphase (Heix et al., 1998). UBF is also inactivated by phosphorylation during metaphase (Klein and Grummt, 1999). Interestingly, SL1 is re-activated in early G1 while UBF remains inactive, suggesting a role for SL1 in addition to rRNA transcription. Therefore, it is possible that AEP/SL1-dependent RNAP2 activation starts in early G1 phase. Unlike many sequence-specific transcription factors, MLL is tethered to chromatin during mitosis, setting the stage for efficient transcription in the next early G1 phase (Blobel et al., 2009). Perhaps transcription of those MLL target genes starts as soon as the next G1 begins, potentially explaining the preference for AEP components as MLL fusion partners. Such biological property may be suited for persistent transcriptional activation of HSC programs genes by the MLL/AEP axis. Given the important roles of SL1 in RNAP1-dependent transcription, gene knockout technologies are not applicable to address these questions. Emerging rapid degradation technology used in the studies of essential factors for viability (Baptista et al., 2017; Warfield et al., 2017) may need to be applied for SL1 components to provide further insights. Because MLL fusion proteins heavily rely on AEP/SL1-dependent gene activation, compounds that inhibit this gene activation process could be used as drugs to treat MLL-r leukemia patients.

## Concluding Remarks

In conclusion, accumulating evidence indicates that RNAP2-dependent transcription mediated by SL1 is a central mechanism used by MLL fusion proteins. However, much of its molecular mechanism remains undocumented and needs to be investigated. Hopefully, precise mechanisms of transcriptional activation by AEP and SL1 will be revealed in greater detail over the next decade.

## Author Contributions

The author confirms being the sole contributor of this work and has approved it for publication.

### Conflict of Interest Statement

The author declares that the research was conducted in the absence of any commercial or financial relationships that could be construed as a potential conflict of interest.
